# The Relationship Between Men’s Self-Perceived Attractiveness and Ratings of Women’s Sexual Intent

**DOI:** 10.3390/bs15081101

**Published:** 2025-08-14

**Authors:** Peter O. Rerick, Tyler N. Livingston, Jonathan Singer

**Affiliations:** 1 Department of Psychology, University of Central Oklahoma, Edmond, OK 73034, USA; 2 Department of Psychology, Angelo State University, San Angelo, TX 76909, USA; tyler.livingston@angelo.edu; 3 Department of Psychological Sciences, Texas Tech University, Lubbock, TX 79409, USA; jonsinge@ttu.edu; 4 Department of Pharmacology and Neuroscience, School of Medicine, Texas Tech University Health Sciences Center, Lubbock, TX 79430, USA; 5 Garrison Institute on Aging, Texas Tech University Health Sciences Center, Lubbock, TX 79413, USA

**Keywords:** sexual arousal, sexual misperception, overperception bias, human mating

## Abstract

Sexual and romantic partners tend to match on various dimensions of mate value including physical attractiveness. Men may be motivated to inflate their self-perceived physical attractiveness to justify pursuing highly attractive women. In the present research, heterosexual men (*N* = 180) received random assignment to a two-way between-participants factorial design that tested the effects of a woman’s physical attractiveness (low vs. high) and the recipient of her ambiguous sexual behavior (the participant himself or another man) on men’s ratings of her sexual intent. Participants rated that attractive women had greater sexual intent compared to unattractive women, but only when the participant himself was the recipient of women’s behavior. Men’s self-perceived physical attractiveness did not vary as a function of the woman’s physical attractiveness except when another man was the recipient of a physically attractive woman’s behavior, which reduced men’s perceptions of their physical attractiveness. Findings suggested that men’s self-perceptions and women’s appearance may bias men’s sexual judgment.

## 1. Introduction

In the first lab demonstration and eventual publication of men’s tendency to infer sexual interest in women’s behavior when none is truly present (sexual overperception), Dr. Antonia Abbey (1982) shared a personal anecdote in which she and a few of her female friends struck up a friendly conversation with two men at a bar between songs the band was playing. Their friendliness ultimately resulted in them excusing themselves from their table to avoid an awkward scene after realizing the men had badly misinterpreted her and her friends’ intentions. Many women have similar stories of men misperceiving friendliness for sexual interest, some of which might explained by error management theory. Error management theory (Haselton & Buss, 2000) suggests that because of the differential reproductive costs of making a mating mistake, men and women most strongly guard against different mating mistakes. Women tend to underperceive men’s interest in long term commitment to avoid the costly mistake of having a child with a man who will not invest resources (e.g., time, food, protection from danger) in her or her offspring (Haselton, 2007; Haselton & Buss, 2009). Because men’s minimum parental investment is much lower than women’s (one of many million sperm cells versus 9 months of gestation and years of breastfeeding, (Buss, 2017)), men face little evolutionary punishment for making a bad short term mating choice. However, the possible cost for missing out on a willing short term sex partner is much higher. As a result, men tend believe that women’s behavior is more indicative of sexual interest than women report it truly being ((Abbey, 1982; Haselton, 2003; see Haselton & Buss, 2000) for an overview of error management theory).

### 1.1. Men’s Overperception of Women’s Sexual Interest

In addition to a basic sex difference in perception of sexual interest, women’s behaviors and physical appearance also influence men’s perceptions of their sexual interest. Behavioral factors such as wearing revealing clothing (Abbey et al., 1987; Koukounas & Letch, 2001; Wade et al., 2021), wearing the color red (Prokop & Pazda, 2016), or using makeup to enhance facial appearance (Cash et al., 1989; Cloud & Perilloux, 2022) all further increase men’s perception of women’s sexual interest. Women commonly report using these and other tactics with the intention to attract sexual partners (Buss, 2006; Schmitt & Buss, 1996), possibly explaining why men tend to believe they are indicative of sexual interest. Other nonbehavioral factors, such as women’s body odor (See Haselton & Gildersleeve, 2011, for review) also appear to influence men’s perceptions of women’s sexual interest. However, these nonbehavioral factors have received comparatively little attention.

### 1.2. Sexual Arousal and Overperception

Men’s own sexual arousal also seems to influence their perceptions of women’s sexual interest. Men’s sexual arousal seems to direct their perception and interpretation of women’s behavior in a way that makes their motivational goal (a sexual encounter) more likely (Maner et al., 2005). Compared to nonaroused men, sexually aroused men rate women’s faces as more attractive (Ditto et al., 2006; Stephan et al., 1971), report greater willingness to engage in sex with partners who would be considered traditionally undesirable (Ariely & Loewenstein, 2006), and report greater willingness to use coercion to obtain sex (Bouffard & Miller, 2014). In more recent experiments, sexually aroused men inferred greater sexual interest in response to various behaviors women might perform compared to nonaroused men (Livingston et al., 2022; Rerick et al., 2020). Given arousal’s influence on men’s perception, the current research tested its role in the perception of the behavior of attractive versus unattractive women.

In many sexual perception studies, men interpret the behavior of a woman towards “a man” in the broad, nonspecific sense; that is, either an unknown hypothetical person or themselves (e.g., Rerick et al., 2020; Perilloux et al., 2012; see Farris et al., 2008 for a review of misperception studies). Comparisons between perception of behavior directed at men themselves versus this nonspecific man are largely lacking. One study did manipulate the target of the behavior, meaning men evaluated behavior from an attractive or unattractive woman performed towards either a hypothetical man, or themselves. Two mediation models indicated that while the attractiveness manipulation did not influence men’s perception of sexual interest when they evaluated behavior towards another man, the manipulation did increase their perceptions of sexual interest when the behavior in question was performed towards themselves. The mediation analyses indicated that sexual arousal was the mediating mechanism, men because more sexually aroused interpreting the behavior of an attractive woman compared to an unattractive woman, but only when the behavior was performed towards themselves (Rerick & Livingston, 2022). The current study aimed to replicate and extend Rerick and Livingston (2022) by adding another potential mediator, men’s perceptions of their own physical attractiveness

### 1.3. The Potential Role of Men’s Attractiveness

Both men and women appear to somewhat constrain their potential mating pool to those who are similar in physical attractiveness to themselves (Berscheid et al., 1971; Folkes, 1982; Kalick & Hamilton, 1986), although some research has resulted in null effects (e.g., Brislin & Lewis, 1968; see Taylor et al., 2011 for review). Some evidence indicates this matching pattern even exists in platonic same sex relationships (Cash & Derlega, 1978). In a series of four studies, Taylor et al. (2011) demonstrated that people’s self-assessments of their sexual desirability predicted their dating matching, that romantic partners are often matched on sexual desirability, and that individuals readily selected partners of similar sexual desirability to their own rather than seeking a maximally desirable partner. For men, wasting mating attempts on women who are unlikely to acquiesce to their solicitations results in emotionally painful as well as socially costly rejection, in addition to possibly wasted economic resources (Buss & Shackelford, 2008). Therefore, despite most men being attracted to very attractive women, it is more reproductively efficient to focus their mating efforts on women who are more likely to reciprocate (i.e., women similar in sexual desirability to themselves). The current study tested whether men would increase their self-ratings of one dimension of sexual desirability, physical attractiveness, to increase their potential mating pool.

In summary, people tend to perceive the beliefs and behaviors of others as consistent with their own goals (Maner et al., 2005), including goals to engage in sexual behavior (Bhatia & Loewenstein, 2022; Loewenstein, 1996). Sexually aroused men interpreting an attractive woman’s behavior might report inflated self-perceived attractiveness to “match” that of the woman (Taylor et al., 2011) to cognitively justify sexual pursuit of more attractive women.

## 2. Method

This study used a 2 (attractiveness: attractive versus unattractive) × 2 (target of behavior: themselves versus another man). First, we hypothesized that men would report greater self-perceived physical attractiveness in response to interpreting an attractive woman’s behavior, but only if the behavior was directed toward themselves versus another man. Second, we hypothesized that men’s self-perceived attractiveness would be positively related to perceptions of women’s sexual interest, regardless of whether women’s behavior was directed toward the man himself or another man. Third, we hypothesized that men would report greater sexual interest from physically attractive versus unattractive women when the women’s behavior was directed toward the man himself, but not when the women’s behavior was directed toward another man, and that this effect would be mediated by self-reported sexual arousal, replicating prior findings (Rerick & Livingston, 2022). The study received research ethics approval from a university in the southern United States. All experimental stimuli and measures as well as data from this study are available on the Open Science Framework (DOI 10.17605/OSF.IO/Y83ZD) (see Supplementary Materials).

### 2.1. Participants

Participants were university students who completed the study online for course credit. The initial sample consisted of 211 participants. Because the hypotheses of this study concerned heterosexual men, we excluded 26 participants from the sample because they failed to identify as predominately heterosexual (Kinsey et al., 1948). We excluded five additional participants who self-reported that they did not pay full attention to the study materials. The final sample contained 180 participants (*M*age = 19.93 years, *SD* = 2.94; 59.4% White).

### 2.2. Materials

Demographic questions included sex, age, and race, and sexual orientation. We used the Kinsey scale (Kinsey et al., 1948; see also Drucker, 2012; Galupo et al., 2014) to assess sexual orientation. Participants responded to instructions to “select the response that best describes your sexual preferences” using a scale from 1 (*exclusively heterosexual*) to 7 (*exclusively homosexual*).

Participants viewed a headshot of either an attractive or unattractive woman referred to in survey materials as Megan. The photos were from the Chicago Face Database (Ma et al., 2015). Prior testing indicated that participants rated both faces as similar in age and race to avoid potential confounds, but different in physical attractiveness to facilitate an effective manipulation^1^. In both photos, the woman was smiling with her lips closed looking into the camera. One sentence below the photo described Megan as a 21-year-old American and provided no other information about her. Participants were assigned at random to view a headshot of either an attractive woman or an unattractive woman and answered a question asking, “How attractive do you find Megan?” with response options from 1 (*Not at All Attractive*) to 7 (*Very Attractive*).

This study used a modified version of the Sexual Intent Perceptions Questionnaire (SIP-Q) from past research (Livingston & Davis, 2020; Livingston et al., 2022; Rerick et al., 2020). The measure contained 25 items designed to capture men’s interpretations of the sexual intent associated with various women’s behaviors and has been highly reliable in past research (α = 0.92; Rerick et al., 2020). In the present study, participants received instructions that varied as a function of experimental condition. In the condition where the woman’s behavior was directed at the men themselves, the instructions read, “Imagine that Megan engages in each of these behaviors with you. Then, indicate how likely it is that this behavior means she wants to have sex with you.” Each behavior had response options that ranged from 1 (*This behavior does NOT AT ALL mean she wants to have sex)* to 7 (*This behavior DEFINTELY means she wants to have sex*). Example items included “She gives you her phone number” and “She does not resist when you initiate intercourse”. In the condition where another man was the target of the woman’s behavior, the instructions read, “Imagine that Megan engages in each of these behaviors with a man. Then, indicate how likely it is that this behavior means she wants to have sex with that man.” For this condition, participants answered the same 25 items as were in the other condition, but modified to change the target of behavior. For example, “She gives a man her phone number” and, “She does not resist when a man initiates intercourse.” Response options for this condition were identical and the behaviors were presented in the same order.

After responding to the above items, participants self-reported their present-state sexual arousal and their self-perceived physical attractiveness. To assess sexual arousal, participants responded to a single item, “How sexually aroused are you?” with response options from 1 (*not at all aroused*) to 7 (*extremely aroused*). To assess self-perceived physical attractiveness, participants responded to a single item, “How physically attractive do you think you are?” with response options from 1 (*not at all attractive*) to 7 (*very attractive*).

### 2.3. Procedure

Participants who indicated their informed consent to proceed first responded to demographic items. Next, participants viewed a photo of Megan according to random assignment. Then, participants responded to the SIP-Q to measure their perceptions of Megan’s sexual intent based on behaviors directed toward the participant himself for another man according to random assignment. The photo of Megan remained visible to participants as they responded to each item on the SIP-Q. Participants then self-reported their present state sexual arousal and self-perceived physical attractiveness. Finally, participants received a debriefing statement describing the nature of the study and redeemed their participation credit.

### 2.4. Statistical Power

An a priori power analysis performed using G*Power version 3.1.9.2 (Erdfelder et al., 1996) that assumed a small to medium effect size of *f*^2^ = 0.125 (Cohen, 2013) indicated 92 participants would be necessary to achieve statistical power of 1 − β = 0.80 for multiple regression analyses. We based our estimate of *f*^2^ = 0.125 on the *R*^2^ values originally reported in prior research (Rerick & Livingston, 2022; *R*^2^ = 0.15 and *R*^2^ = 0.26 for the “a man” and “yourself” conditions, respectively).

## 3. Analysis Plan and Results

**Figure 1:**
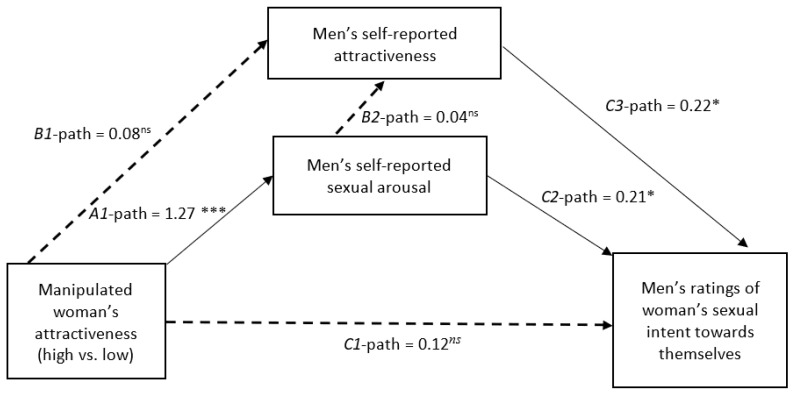
Mediation analysis depicting the effect of target attractiveness on men’s ratings of women’s sexual intent through self-reported sexual arousal and attractiveness. In this analysis, participants imagined that they were the target of the woman’s behavior. Note. *** = *p* < 0.001, * = *p* < 0.05, *ns* = nonsignificant. Path coefficients represent unstandardized beta scores. The unattractive condition served as the reference group. The *C1* path represents the direct effect of the manipulation accounting for both mediators.

**Figure 2:**
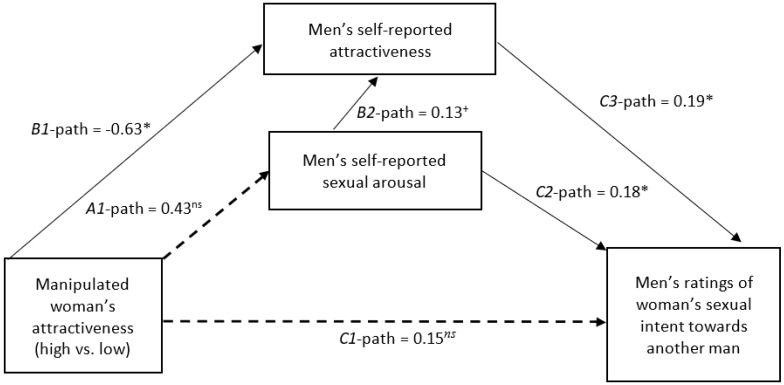
Mediation analysis depicting the effect of target attractiveness on men’s ratings of women’s sexual intent through self-reported sexual arousal and attractiveness. In this analysis, participants imagined that another man was the target of the woman’s behavior. Note. * = *p* < 0.05, + = *p* < 0.10, *ns* = nonsignificant. Path coefficients represent unstandardized beta scores. The unattractive condition served as the reference group. The *C1* path represents the direct effect of the manipulation accounting for both mediators.

We assessed the effectiveness of the attractiveness manipulation, its direct effect on our two composite measures, and the reliability of our two composite measures. Next, we assess whether our manipulation affected sexual arousal similarly in both target of behavior conditions. For the primary analyses, each dependent variable was assessed separately using two identical mediation procedures. We attempted to fit the same model as Rerick and Livingston (2022) but added attractiveness as a potential mediator (see Figure 1 and Figure 2) using Lavaan (version 0.6-19, Rosseel, 2012) in RStudio (version 2025.05.0.496, Mariposa Orchid, Posit Team, 2025). Each mediation model contained three regression equations. The first regression equation (model 1) used the attractiveness manipulation^2^ (A1) to predict the measure of self-reported arousal. The second equation (Model 2) used the attractiveness manipulation (B1) and self-reported arousal (B2) to predict self-perceived attractiveness. The final equation (Model 3) used the SIP-Q as the dependent variable, and the attractiveness manipulation (C1), self-reported arousal (C2), and self-perceived attractiveness (C3) as predictors. Each mediation model was bootstrapped to 10,000 iterations.

### 3.1. Physical Attractiveness Manipulation Check

A *t*-test indicated the attractiveness manipulation was effective (*t*(178) = 14.22, *p* < 0.001, *d* = 2.12). Men who viewed the attractive photo of Megan rated her as significantly more attractive (*M* = 4.21, *SD* = 1.50) than men who viewed the unattractive photo of Megan (*M* = 1.54, *SD* = 0.95).

### 3.2. Reliability

We combined all 25 items of the SIP-Q from each target condition into two composite variables by computing the mean across all 25 items for each participant. Both Cronbach’s alphas indicated the composite measures were highly reliable (αs = 0.96). See Table 1 for means and standard deviations for all individual items in both target conditions.

### 3.3. Direct Effect of the Attractiveness Manipulation

We used regression to assess the direct of effect of the attractiveness manipulation on men’s perceptions of women’s sexual intent. The attractiveness manipulation did not directly predict men’s interpretation of women’s sexual interest towards other men (*b* = −0.12, *t*(86) = −0.45, *p* = 0.65). It also did not significantly predict men’s interpretation of women’s sexual interest towards themselves (*b* = −0.40, *t*(90) = −1.59, *p* = 0.11) despite trending in the expected direction.

### 3.4. Assessment of Self-Reported Sexual Arousal

We conducted an ANOVA using self-reported sexual arousal as the dependent variable and the attractiveness manipulation and the target of behavior conditions as independent variables. There was a main effect of attractiveness condition (*F*(175) = 11.05, *p* = 0.001, *η_p_*^2^ = 0.06) such that men who viewed the attractive photo of Megan reported significantly more sexual arousal (*M* = 2.72, *SD =* 1.87) compared to men who viewed the unattractive photo of Megan (*M* = 1.87, *SD =* 1.53). There was no main effect of target of behavior condition (*F*(175) = 0.71, *p* = 0.40) and no significant interaction (*F*(175) = 2.67, *p* = 0.10).

### 3.5. Primary Analyses

The first mediation model depicted in Figure 1 tested the effects of the attractiveness manipulation, self-perceived attractiveness, and self-reported sexual arousal on men’s interpretation of women’s behaviors with the participants themselves as the target. Model 1 indicated the attractiveness manipulation (A1-path) significantly increased self-reported sexual arousal (*b* = 1.27, *z* = 3.63, *p* < 0.001, *R*^2^ = 0.03). Model 2 (*F*(2,88) = 0.19, *p* = 0.79, *R*^2^ = 0.004) indicated neither the attractiveness manipulation (B1-path; *b* = 0.08, *z* = −0.24, *p = 0*.81) nor self-reported sexual arousal (B2-path; *b* = 0.04, *z* = 0.35, *p* = 0.72) were significant predictors of self-reported attractiveness. Finally, Model 3 (*F*(2,87) = 6.07, *p* < 0.001, *R*^2^ = 0.17) indicated both self-reported sexual arousal (C2-path; *b* = 0.21, *z* = 2.41, *p* = 0.02) and self-reported attractiveness (C3-path; *b* = 0.21, *z* = 2.20, *p* = 0.03) positively predicted men’s interpretations of women’s sexual interest towards themselves. The attractiveness manipulation (C1-path; *b* = 0.12, *z* = 0.47, *p* = 0.63) was not a significant predictor^3^. The indirect effect of the attractiveness manipulation on sexual interpretations through self-reported sexual arousal was marginally significant (A1 to C2; *b* = 0.26, *z* = 1.85, *p* = 0.06).

The second mediation model depicted in Figure 2 tested the effects of the attractiveness manipulation, self-perceived attractiveness, and self-reported sexual arousal on men’s interpretation of women’s behaviors with another man as the target. Model 1 indicated the attractiveness manipulation (A1-path) did not increase self-reported sexual arousal (*b* = 0.43, *z* = 1.21, *p* = 0.23, *R*^2^ = 0.02). Model 2 (*F*(2,85) = 3.11, *p* = 0.04, *R*^2^ = 0.07) that the attractiveness manipulation (B1-path; *b* = −0.63, *z* = −2.17, *p = 0*.03) significantly decreased self-reported attractiveness. Self-reported sexual arousal (B2-path; *b* = 0.13, *z* = 1.76, *p* = 0.08) was only a marginal predictor of self-reported attractiveness. Finally, Model 3 (*F*(2,84) = 3.83, *p* = 0.01, *R*^2^ = 0.12) indicated both self-reported sexual arousal (C2-path; *b* = 0.18, *z* = 2.37, *p* = 0.02) and self-reported attractiveness (C3-path; *b* = 0.19, *z* = 2.26, *p* = 0.02) positively predicted men’s interpretations of women’s sexual interest towards another man. The attractiveness manipulation (C1-path; *b* = 0.12, *z* = 0.47, *p* = 0.63) was not a significant predictor. There were no significant indirect effects in the second mediation model.

## 4. Discussion

The purpose of the present research was to examine how two factors—women’s physical attractiveness and the recipient of her behavior—can influence men’s perceptions of her sexual intent. Given that women and men often convey sexual intent using behavioral cues (i.e., indirectly) rather than explicit verbal communication (Hickman & Muehlenhard, 1999; McCormick, 1979), incidental features of the communicator, such as her physical attractiveness, may affect how her message is received. Moreover, observers may attribute women’s behaviors differently depending on whether those behaviors are directed toward themselves versus a third party (Jones & Nisbett, 1972; Livingston & Rerick, 2023). The positionality of the observer—that is, whether the target of women’s behavior is the observer himself versus a third party—is relevant in interpersonal and legal contexts. Accurate interpretation of women’s behavioral cues can protect against men’s overperception of women’s sexual intent (Abbey, 1982; Haselton, 2003; Haselton & Buss, 2000), thus preventing unwanted sexual advances that could lead to sexual misconduct. If sexual misconduct allegations arise, the perceptions of third parties such as investigators and jurors can influence legal outcomes. The present findings have implications for psychological research and applications to real-world interpersonal and legal contexts.

Two primary findings replicated across both models represented in Figure 1 and Figure 2 in the present study. First, regardless of whether the target of women’s behavior was the observer himself or a third party, participants’ self-reported sexual arousal was positively associated with their ratings of women’s sexual intent. Consistent with error management theory, men may be prone to interpret ambiguous nonverbal behavior as evidence of women’s sexual willingness to capitalize on a potential mating opportunity (Haselton & Buss, 2000). This effect may be exacerbated by strong affective states such as sexual arousal, which can motivate men’s reasoning in favor of a desired outcome (Kunda, 1990; Maner et al., 2005). The positive association between men’s self-reported sexual arousal and ratings of women’s sexual intent may be a source of sexual miscommunication and thus an intervention target to prevent sexual misconduct (Livingston et al., 2022; Rerick et al., 2022). Second, regardless of whether the target of women’s behavior was the observer himself or a third party, participants’ self-perceived physical attractiveness was positively associated with their ratings of women’s sexual intent, which is a novel finding. More physically attractive persons tend to engage in a greater number of sexual relationships (Rhodes et al., 2005), perhaps in part because people tend to associate physical attractiveness with other positive interpersonal qualities (Dion et al., 1972; Palmer & Peterson, 2021). Given their greater success initiating willing sexual relationships, more physically attractive men may be prone to interpret women’s nonverbal behavior as an expression of genuine romantic interest. Future research should test strategies to counter this bias among physically attractive observers.

Other findings differed between both models. Women’s physical attractiveness affected men’s self-reported sexual arousal when her behavior was directed toward the observer himself rather than toward a third party. Men who were the recipient of a physically attractive woman’s nonverbal behavior reported greater sexual arousal compared to men who were the recipient of a physically unattractive woman’s behavior. However, no such relationship between women’s physical attractiveness and men’s self-reported sexual arousal emerged among men interpreting behavior directed toward a third party. Interactions with physically attractive women may enhance men’s sexual arousal, which may in turn lead men to interpret sexual intent from her behavior (Bouffard & Miller, 2014, 2024; Rerick et al., 2020). Indeed, the present study found evidence of this indirect effect among men who were the target of women’s behavior, although unlike in previous research (Rerick & Livingston, 2022), this indirect effect was only marginally significant in the present study. More physically attractive women may be at increased risk of sexual misconduct due to misinterpretation of their intent. These findings extend prior research suggesting that victim physical attractiveness may be a risk factor for sexual offenses against juveniles (Savolainen et al., 2020). This indirect effect was not present among men interpreting behavior directed toward a third party, suggesting that investigators and jurors may be fairer arbiters of sexual miscommunication.

The effect of women’s physical attractiveness on men’s self-perceived physical attractiveness also differed between the models. Among men reporting on women’s behavior directed toward themselves, there was no association between the woman’s physical attractiveness and men’s self-perceptions. This result was contrary to our expectations of increased self-perceived attractiveness in response to interpreting an attractive woman’s behavior based on the matching hypothesis (Taylor et al., 2011). However, among men reporting on women’s behavior directed toward another man, men observing a physically attractive woman reported lower self-perceived physical attractiveness compared to men observing a physically unattractive woman. The null effect among men reporting on behavior directed toward themselves may suggest generally self-serving perceptions of physical attractiveness, consistent with evidence that people evaluate themselves positively to protect self-esteem (Alicke & Sedikides, 2009; Zell et al., 2020). These generally self-serving perceptions may have led participants to assume that they could receive attention from both physically attractive and unattractive women. Social comparison theory, which suggests that people compare themselves to others to assess their own self-worth (see Gerber et al., 2018), might explain why this effect differed among men who considered women interacting with another man. Upward social comparisons occur when people gauge their worth versus a person of higher status than themselves, whereas downward social comparisons occur when people gauge their worth versus a person of lower status than themselves. Men who considered a physically attractive woman interacting with another man may have engaged in upward social comparison to the man receiving a physically attractive woman’s attention, leading participants to feel comparatively inferior on dimensions such as their own attractiveness. These findings suggest that self-perceptions, which can vary based on social context, might influence attributions of nonverbal behavior. Given the novel finding that men’s self-perceived attractiveness was related to perceptions of women’s sexual interest, any explanation for these results will require several replications before being considered reliable.

Present findings have implications for interpersonal relationships and for the legal system. Men’s self-perceived physical attractiveness might influence their interpretations of women’s behavior, leading to potential misunderstandings of sexual intent. Women’s physical attractiveness may be a risk factor as well, as it may increase men’s sexual arousal and bias their attention toward cues that convey sexual interest (Kunda, 1990; Maner et al., 2005; Rerick et al., 2020). Heightened sexual arousal can impair judgment (Ariely & Loewenstein, 2006) and lead to misattributions of behavior detrimental to interpersonal relationships. Direct verbal communication could help to clarify intentions (Shumlich & Fisher, 2020), especially for men who are more physically attractive and thus perhaps at increased risk for overperceiving women’s sexual interest. Ambiguous behavior can be easily misinterpreted, especially when influenced by factors such as physical attractiveness and sexual arousal. Interpersonal relationships characterized by clear statements of sexual consent versus nonconsent may be beneficial to both parties (Featherstone et al., 2024). Moreover, positionality can influence judgments, as men tend to interpret women’s behavior differently when they are the recipient versus an observer. Intervention from third party bystanders may help to avoid unwanted outcomes associated with misinterpretations of sexual intent (Park & Kim, 2023).

The present study is not without limitations that future research may address. First, although sexual arousal was the hypothesized mechanism linking women’s physical attractiveness to men’s interpretations of their behaviors, this study measured self-reported sexual arousal rather than manipulating it via an experimental paradigm (see Livingston et al., 2022; Rerick et al., 2020, 2022). Future research should examine the extent to which the present effects replicate among participants in a heightened state of sexual arousal, which can function as a drive state that motivates goal-directed behavior (Loewenstein, 1996). Second, the present research manipulated women’s physical attractiveness under the assumption that desirability would bias interpretations of women’s behavior. Although appearance is an important component of desirability, other factors such as humor, intellect, and empathy inform comprehensive evaluations (Edlund & Sagarin, 2014). Future research should manipulate these factors as well, either discretely or in combination, to gain a more nuanced understanding of how women’s characteristics inform men’s interpretations of their behavior. Third, the present research measured both mediators, self-reported arousal and self-reported physical attractiveness, after the dependent measures rather than before. Although we believe we have made a theoretical case for the causal ordering of these variables, the present design and statistics cannot confirm the specific causal order. Previous research does strongly suggest a causal relationship between men’s sexual arousal and increased perception of women’s sexual intent (Rerick et al., 2020), but future research should directly manipulate self-perceptions of physical attractiveness and test its causal effect on men’s perceptions of women’s sexual interest.

## Figures and Tables

**Table 1:** Means and standard deviations for the measure of ratings of sexual intent.

Instructions: Imagine that Megan engaged in each of these behaviors with a man [you]. Then, indicate how likely it is that this behavior means Megan wants to have sex with that man [you].				
**Item (She…)**	**Ratings with Other Man as Target**		**Ratings with Self as Target**	
	***M***	***SD***	***M***	***SD***
…drinks with a man she just met	2.13	1.36	1.88	1.34
…becomes intoxicated with alcohol on a date with a man she has not met before	2.45	1.62	2.04	1.56
…becomes intoxicated with alcohol at a party without a date	2.14	1.51	1.77	1.45
…becomes intoxicated with alcohol at a party and leaves the party with a man she just met	3.65	2.18	2.88	1.99
…goes out to lunch with a man	2.06	1.35	2.19	1.48
…approaches a man to initiate conversation	2.09	1.24	2.17	1.53
…sits or stands close to a man	1.92	1.14	2.26	1.54
…gives a man her phone number	2.91	1.50	2.92	1.62
…wears perfume	2.26	1.53	2.27	1.50
…acts very affectionate toward a man at a party	4.14	1.70	3.80	1.71
…tells a man how great he looks	3.03	1.48	2.93	1.72
…invites a man to her house for dinner	3.40	1.78	3.32	1.87
…Leans up Against him	3.54	1.76	3.88	1.81
…Lets a man perform oral	5.82	1.77	6.01	1.47
…Let’s a man touch her breasts through her clothes	5.63	1.68	5.90	1.46
…takes of shirt and bra around a man	5.22	1.92	5.43	1.70
…goes to a man’s residence during a date to be alone	4.53	1.98	4.20	2.01
…dresses very sexily	4.04	2.21	3.70	2.07
…touches a man’s bare genitals	5.90	1.633	6.22	1.21
…takes off her pants, skirt and underwear	5.69	1.78	5.88	1.57
…spends the night at a man’s residence	4.17	2.03	3.90	1.90
…doesn’t resist when man initiates intercourse	4.95	1.92	5.17	1.84
…sends nude pictures	5.17	1.72	5.55	1.56
…says yes to an invitation to watch a movie at a man’s residence	3.40	1.80	3.36	1.94
…uses marijuana on a date with a man she has not had sex with before	2.38	1.60	2.16	1.51
**Overall *M* and *SD***	3.67	1.24	3.71	1.22

Note. Item wording varied slightly depending on experimental condition.

## Data Availability

Data supporting the results presented here can be found on the Open Science Framework (https://doi.org/10.17605/osf.io/y83zd, accessed on 9 August 2025).

## Notes

1 According to the Chicago Face Database, the attractive photo was rated by 94 people who estimated the age of the woman was 23.96 years. Just over 97% of raters perceived the woman as White. The unattractive photo was rated by 99 people who estimated the age of the woman was 24.95 years. Over 98% of these raters perceived the woman as White. The attractive photo was received an attractiveness rating of 4.75/7. The unattractive photo received an attractiveness rating of 1.61/7. We informed participants the women were 21 years old to aid the impression they would be realistic romantic or sexual partners for the typical college aged male.

2 The attractiveness manipulation is dummy coded using the unattractive condition as the reference group, so positive betas mean a given dependent variable is higher for the attractive face compared to the unattractive face.

3 Even without a significant direct effect, a significant indirect is possible and would indicate a mediated effect (See Zhao et al. (2010) for a nontechnical discussion, but see (Kenny et al., 1998; MacKinnon & Fairchild, 2009) for more technical discussions).

## Supplementary Materials

All experimental stimuli and measures from this study are available on the Open Science Framework (https://doi.org/10.17605/OSF.IO/Y83ZD, accessed on 9 August 2025).

## Abbreviations

The following abbreviations are used in this manuscript:

SIP-Q	Sexual Intent Perceptions Questionnaire
